# Magnetic moment inversion at giant flux jump: dynamical property of critical state in type-II superconductors

**DOI:** 10.1038/s41598-019-42699-5

**Published:** 2019-04-17

**Authors:** Viktor Chabanenko, Adam Nabiałek, Roman Puźniak, Olena Kuchuk, Oleksandr Chumak, Felipe Pérez-Rodríguez, Umapada Pal, Valentin Garcia-Vazquez, Raul Cortés-Maldonado, Jun Qian, Xin Yao, Henryk Szymczak

**Affiliations:** 1 O. Galkin Donetsk Institute for Physics and Engineering, NASU, Pr. Nauki 46, 03680 Kyiv, Ukraine; 2 Institute of Physics, Polish Academy of Sciences, Aleja Lotników 32/46, PL-02668 Warsaw, Poland; 3 Instituto de Física, Benemérita Universidad Autónoma de Puebla, Apdo. Post. J-48, Puebla, Pue. 72570 Mexico; 4 Departamento de Ciencias Básicas, Tecnológico Nacional de México, Ave. Instituto Tecnológico s/n, Apizaco, Tlaxcala CP 90300 Mexico; 5 School of Physics and Astronomy, Shanghai Jiao Tong University, 800 Dongchuan Road, Shanghai, 200240 China

**Keywords:** Superconducting properties and materials, Engineering, Energy science and technology

## Abstract

Experimental evidence of tremendous magnetic moment dynamical inversion, from metastable trapping state to the state with essentially the same moment oriented in the opposite direction, appearing during giant flux jump connected to thermomagnetic avalanche process in superconducting YBa_2_Cu_3_O_7−*δ*_ single crystal, is presented. Magnetization inversion takes place in the system, without thermal contact between sample and sample holder, with a tremendous stored energy once the avalanche process is completed in quasi-adiabatic conditions. A model of magnetic moment inversion, caused by the jump between two metastable states of superconductor with the same energy storage, is presented and discussed in terms of the critical state with peculiar evolution of the critical-current spatial distribution. Importantly, knowledge of conditions of the appearance of such a phenomenon is crucial for applications of bulk superconductors as “permanent” magnets, for example, in superconducting levitation devices, etc.

## Introduction

The magnitude of the critical current density, *J*_c_, is a measure of the inhomogeneity of magnetic field distribution, caused by pinning of the magnetic flux in an external magnetic field *H*. In original Bean’s critical state model, *J*_c_ is assumed to be a constant, independent on magnetic field^1^. In Kim, Hempstead, and Strnad^2,3^ and Anderson^4^ approach, it is assumed that the critical current density is inversely proportional to the local magnetic field, *J*_c_ ~ 1/(*B* + *B*_0_), where *B* is the magnetic field induction and *B*_0_ is constant. Such critical state model allows to describe the magnetic properties of hard type-II superconductor and to calculate, for example, magnetization^5–7^ or flux-pinning-induced magnetostriction^8–10^. One of the widely developed concepts in highly anisotropic high-*T*_c_ materials is the nature of the vortex state and pinning that is at the root of the critical sate model^11^. Relaxation of the metastable critical state through a jump of the magnetic flux, ΔΦ_av_, is known for more than half a century^3,12,13^ and it is described by the Maxwell and the heat balance equations^14,15^. The speed of this process is limited by the dissipative losses that occur during an avalanche. The jumps are observed most often during the change of magnetic field and they are usually partial or, less often, complete. So called giant flux creep must be considered in stabilizing against flux jumps^16^. The partial jumps arise when the avalanche embraces only some limited portion of the superconductor volume. If the magnetic moment decreases to almost zero, the jump is assumed to be complete. A sharp reduction of sample magnetic moment to zero due to the avalanche has rarely been observed experimentally, and a slight change in the sign of the moment was treated as an exotic exceptional fact^17^.

Superconducting YBa_2_Cu_3_O_7−*δ*_ is the most well-known high-*T*_c_ superconductor. Pinning sites of this superconductors have been studied in much detail for melt-process melt grown YBa_2_Cu_3_O_7_^18^. The experimental evidence of magnetic moment inversion in superconducting YBa_2_Cu_3_O_7−*δ*_ single crystal, without energy *E*_trn_ dissipation connected with the moment turning at zeroing magnetic field $({E}_{{\rm{trn}}}\sim \overrightarrow{M}\cdot \overrightarrow{H})$, is presented in this letter. However, it should be noted that the temperature of the superconductor changes locally during an avalanche of magnetic flux. Direct contact measurements, for example, using a thermocouple, confirm this. At least two mechanisms of energy dissipation accompanying the motion of the Abrikosov vortex as an element of the magnetic flux of a superconductor are well known. These are resistive energy losses in the normal vortex core and two phase transitions at the front and backside of the vortex core. The first one is the transition from the superconducting to the normal state and the second one, reverse, from normal to superconducting state. The observation presented here injects new dynamical feature of critical state of hard type-II superconductors: the jump transition from one branch of hysteresis loop in flux trapping regime to another one, i.e. jump from superconducting magnetic state characterized by magnetization value +*M* to that one characterized by −*M* or vice versa. The process is quite similar to well-known thermodynamic jumps between different metastable states in ferromagnets or ferroelectrics. However, the Gibbs energies in the initial and final states are not equal but differ by $\sim \,\overrightarrow{M}\cdot \overrightarrow{H}$. Though this difference could be negligible at external magnetic field close to zero, the extension of the instable magnetization loop region clearly reveals the role of the external field as a driving force of the flux avalanche. We propose a mechanism for the magnetic moment inversion. Blocking of such inversion is crucial for the application of bulk superconductors as “permanent” magnet in power systems^19–21^.

## Experimental Details

**Figure 1:**
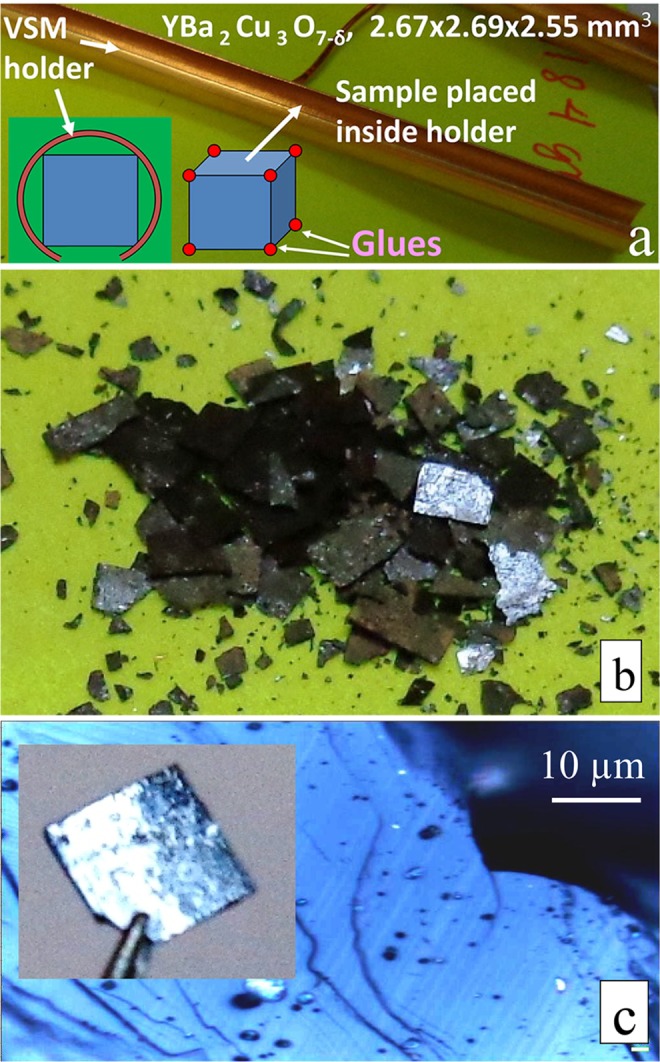
(**a**) Visualization of installation of the sample in the holder, to be fixed into vacuum chamber, to avoid heat flows between the sample and the surrounding media. The cross section of the holder with the sample is shown at the bottom left. (**b**) Images of magnetolaminated crystal, and (**c**) twin boundaries at the surface of laminar crystals.

A relatively large YBa_2_Cu_3_O_7−*δ*_ single crystal of 2.67 × 2.69 × 2.55 mm^3^ and 0.1184 g mass was utilized for magnetic studies. The single crystal was grown by top-seeded solution growth^22^. The as-grown sample became superconducting as a result of oxygenation at 500 °C for 72 h. Since a normal oxygenation method has been applied, the twin structure, due to a tetragonal to orthorhombic transition, was present in the crystal. Magnetic properties of the sample were examined in a Quantum Design PPMS DynaCool-9 System equipped with vibrating sample magnetometer (VSM) option. The crystal was placed into a special holder, i.e., on the cut of gold-coated brass tube (Fig. 1(a)). Figure 1(a) shows the exact placement of the sample inside the holder (see cross section of the sample inside the holder). Such a fastening excludes the reversal of the sample in a magnetic field. The size of the diagonal on the edge of the sample was slightly larger than the internal diameter of the tube holder. The sample was inserted into the tube through the holder’s open end. The sharp edges of the cube of the studied sample only were in mechanical contact with the holder. It practically excludes the possibility of heat flow between the sample and the sample holder, due to limited thermal contact. Holder with sample was installed in a vacuum chamber to reduce heat flows and to limit the dissipation of energy. The residual pressure of He gas in the chamber was equal to about 38.4 Torr. The magnetic field was applied along to the crystallographic *c*-axis of the crystal. The critical current density *J*_c_, estimated from first flux jump field, was ≥ 3 · 10^9^ A/m^2^ at *T* = 2 K.

It is well known for a long time that, the repeated cycling of a strong magnetic field at liquid helium temperature, under conditions of magnetic flux jumps during thermomagnetic avalanches, can lead to microcracking damage of the crystal^23,24^ or to the magnetolamination phenomenon. Magnetolamination, explained in terms of the motion of twin boundaries, occurring under mechanical loading of the sample, was reported already in textured YBa_2_Cu_3_O_7−*δ*_ superconductor^25^ and was observed in the studied single crystal too (Fig. 1(b,c)). This phenomenon consists in mechanical lamination parallel to the *ab*-plane into thin platelets, due to pressure generated by strong magnetic field. A magnetic field as high as 8 T at 4.2 K can produce a pressure ~ 0.5 kbar on the sample. Because of the microcracking of the crystal and the phenomenon of magnetolamination in a strong magnetic field, it was impossible to repeat hysteresis loop measurements performed at the same conditions for the same crystal. Nevertheless, magnetolamination allowed us to observe the surface structure of the inner layers of the studied crystal. Figure 1(c) shows the surface of one of the thin plates. A clear grid of parallel lines, which are the twinning surfaces that have come out of the bulk crystals, is visible.

## Results and Discussion

**Figure 2:**
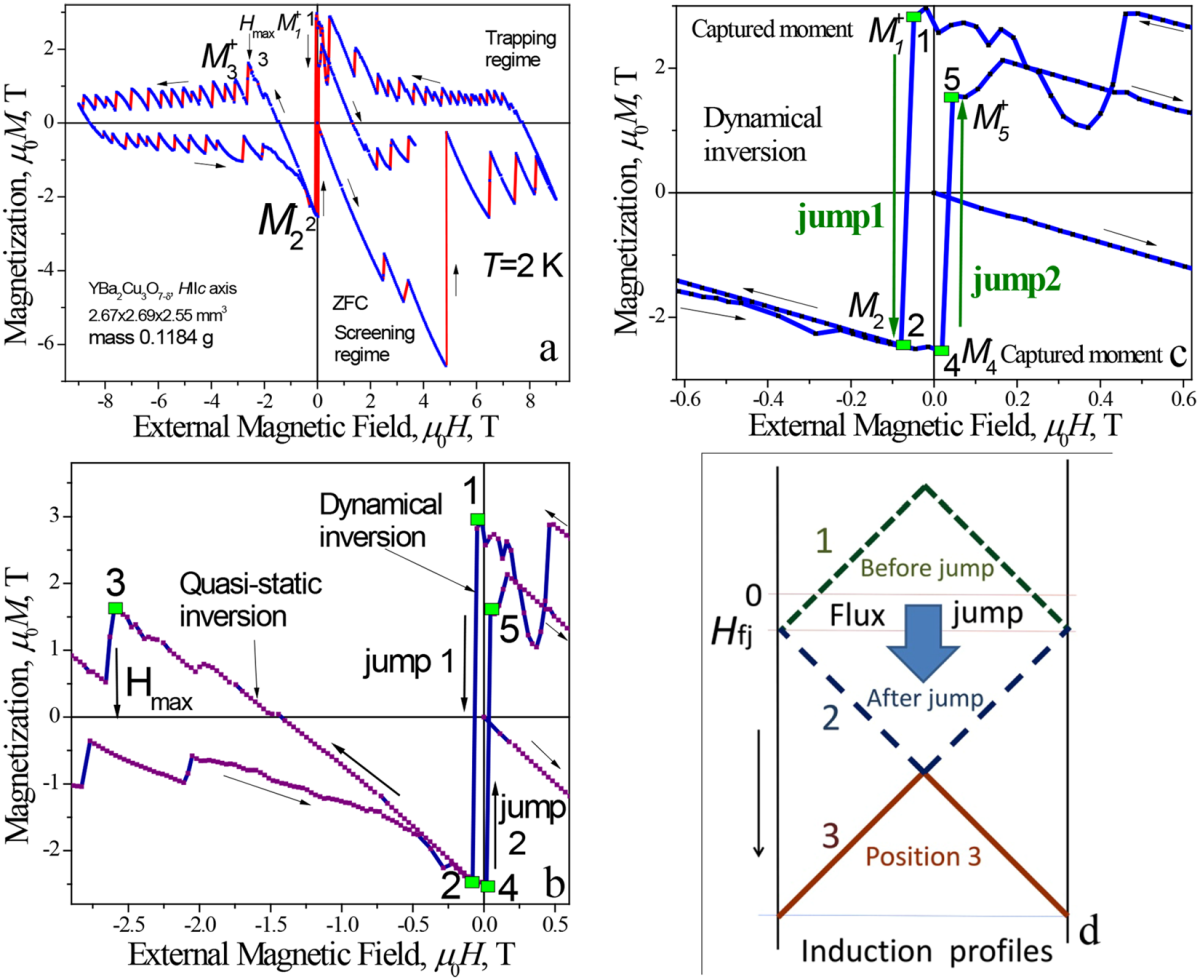
(**a**) Remagnetization loop *M*(*H*) for the YBa_2_Cu_3_O_7−*δ*_ single crystal at *T* = 2 K under a magnetic field *H* || *c*-axis. (**b**) Extension of the data presented in panel (a) at the vicinity of positions 1 in *M*(*H*) loop, i.e., for the field range below the appearance of jump 1; at the vicinity of position 2, i.e., for the field range just above dynamical inversion of magnetic moment, appearing during jump from position 1; and at the vicinity of position 3, i.e., for the field range above quasi-static inversion. (**c**) Extension of the loop in the vicinity of dynamical inversion of giant magnetic moment around *H* = 0. (**d**) Profiles of magnetic induction for different spots (positions 1, 2, 3) of hysteresis loop (**b**).

Figure 2(a) shows the hysteresis loop measured at *T* = 2 K, while panels (b) and (c) are focused on its details. The loop was recorded at the first remagnetization cycle under the influence of increasing and then decreasing magnetic field. In the virgin diamagnetic part of the magnetization curve, i.e., in the first quarter - screening mode - a tremendous thermomagnetic avalanche, following two partial flux jumps, is visible at around *μ*_0_*H* = 4.85 T. The flux jump is an almost complete, i.e., equal to about 96% of initial magnetization value. Then, the typical flux jump behavior of magnetization was observed up to 9 T^17^. Many small jumps appeared when the magnetic field was decreased to *H* = 0, i.e., in the second quarter of the loop, presented in Fig. 2, in trapping mode.

It is known that, at a negative field (the third quarter) the trapped magnetic moment of a bulk sample reaches a maximum. In this region, the largest induction gradient arises, determining the place of the highest instability of critical state on the magnetization loop^6^. As a rule, the flux avalanche in this place is the largest in magnitude. In the studied crystal, a dramatic event occurred when the direction of the magnetic field was reversed (Fig. 2(a,b,c)). The duration of such process was estimated to be equal to several tens of millisecond.

Figure 2(b,c) shows details of the remagnetization process in the vicinity of zero field. As can be seen, the magnetization associated with the trapped magnetic flux is inverted during the thermomagnetic avalanche at *μ*_0_*H*_fj_ = −0.048 T. Such behavior can be classified as dynamical inversion. The magnetization jumps from the state with a positive ${M}_{1}^{+}$ (denoted as 1 in the figure, ${\mu }_{0}{M}_{1}^{+}$ = 2.82 T) to the state with a negative value of magnetization, ${\mu }_{0}{M}_{2}^{-}$ equal to −2.46 T (Fig. 2(c)). The appearance of negative magnetization ${M}_{2}^{-}$ could be explained by inversion of critical current (current flowing in opposite direction).

A subsequent slow increase of the field in the negative direction leads to a gradual magnetization reversal and a coming back from the state ${M}_{2}^{-}$ (position 2 in the figure, the fourth quarter on the *M*(*H*) curve) to the third quarter to the ${M}_{3}^{+}$ state in the magnetic field of ${\mu }_{0}{H}_{{\rm{\max }}}$ = −2.6 T. During this process, the magnetization of the studied sample was completely reversed (quasi-static transition ${M}_{2}^{-}\to {M}_{3}^{+}$), i.e., the quasi-static reversal of the induction profile (from position 2 to position 3, Fig. 2(d)) occurred. Quasi-static inversion of the profile allowed us to estimate the value of critical current density. As follows from Fig. 2(d), field ${\mu }_{0}{H}_{{\rm{\max }}}\approx 2{B}_{{\rm{p}}}$, where the full penetration field ${B}_{{\rm{p}}}=\frac{1}{2}{\mu }_{0}{J}_{{\rm{c}}}\,d$, and *d* is the thickness of the sample. The value of the critical current density *J*_c_ determined with relation for *B*_p_ was ≈1 · 10^9^ A/m^2^, being in good agreement with the data obtained from the width of the hysteresis loop.

As the field decreased from the maximum value in the fourth quarter, the magnetization curve *M*(*H*) repeated the behavior observed in the second quarter (Fig. 2(a)), what is typical for remagnetization process. When the field approached the zero value, the trapped magnetization became comparable in the value to the ${M}_{2}^{-}$, magnetization obtained as a result of avalanche inversion. This indicates that, a dynamical inversion of the magnetization of the superconductor, i.e., magnetic flux jump, from the metastable position 1 on the hysteresis loop *M*(*H*) in the third quarter to the metastable position 2 located in the fourth quarter, actually occurred as a result of the thermomagnetic avalanche.

On switching the direction of the external field, the dynamical inversion of the trapped magnetization (Fig. 2(c), jump 2) was repeated, i.e., a jump of the magnetization: ${M}_{4}^{-}\to {M}_{5}^{+}$ was observed. In this case, the value of the inverted magnetization ${M}_{5}^{+}$ differed slightly from the value of the maximum magnetization trapped in the second quarter. This might be due to instability of the trapped flux in the crystal in this field region. Such an instability was observed already in the second quarter, when the field was scanned from *H* = +9 T to zero.

To observe the inversion of the induction profile in the entire sample - the phenomenon presented in the manuscript - several conditions must be fulfilled:

- The large size of a single crystal is required (for the appearance of avalanches),
- experiment must be performed at low temperatures (high critical current required),
- experimental conditions must be close to adiabatic.

It is known that, in order to remagnetize large size crystals and to use them as permanent magnets, a metal band must be worn on the samples to prevent the destruction of the crystal during the very first magnetizations. In our case, the requirement of keeping experimental conditions close to the adiabatic ones excluded the bandage. Thus, it was impossible to effectively repeat the sample remagnetization, since, the crystal was cracked; thermomagnetic avalanches became weak due to diminishing crystal size or they did not occur at all. We must clearly state that the best picture, which so far was managed to observe, is presented in the manuscript.

The cracks arise due to jumps of mechanical stresses (*P*) arising due to jumps of magnetic flux^25,26^. By the order of the magnitude the maximum pressure in the sample is^25^ *P* ≈ Δ*B*(*B*_ext_ − Δ*B*/2)/*μ*_0_, where Δ*B* = *B*_ext_ − *B*_int_, *B*_ext_ is the induction of external magnetic field and *B*_int_ is the induction inside of superconductor. Substituting the typical values Δ*B* = 4 T and *B* = 6 T we obtain that in the region of magnetic-flux jump the characteristic pressure value in *ab*-plane is *P* = 10–20 MPa. This additional stresses could be responsible for individual cracks. It has been proved in a number of experiments that the twin boundaries become mobile at stress of 5–10 MPa. These stresses could be responsible for lamination.

Let us consider the process of a thermomagnetic avalanche in the superconductor. The investigation of induction change, occurring as a result of the flux avalanche on the surface of the superconductor, performed with the help of magneto-optical^27–29^ or the Hall sensor array measurements^30–32^, in the screening mode (ZFC measurement), revealed local formation of a “dome-like” profile of induction. In such case, the penetration of a flux avalanche results in the appearance of paramagnetic circular current in the diamagnetic region of a superconductor, that determines the local inversion of the magnetic induction profile (Fig. 26 in^33^). On the other hand, in the case of an avalanche exit, i.e. in the flux trapping regime, a circular diamagnetic current appears in the paramagnetic region of the superconductor (Fig. 27 in^33^).

**Figure 3:**
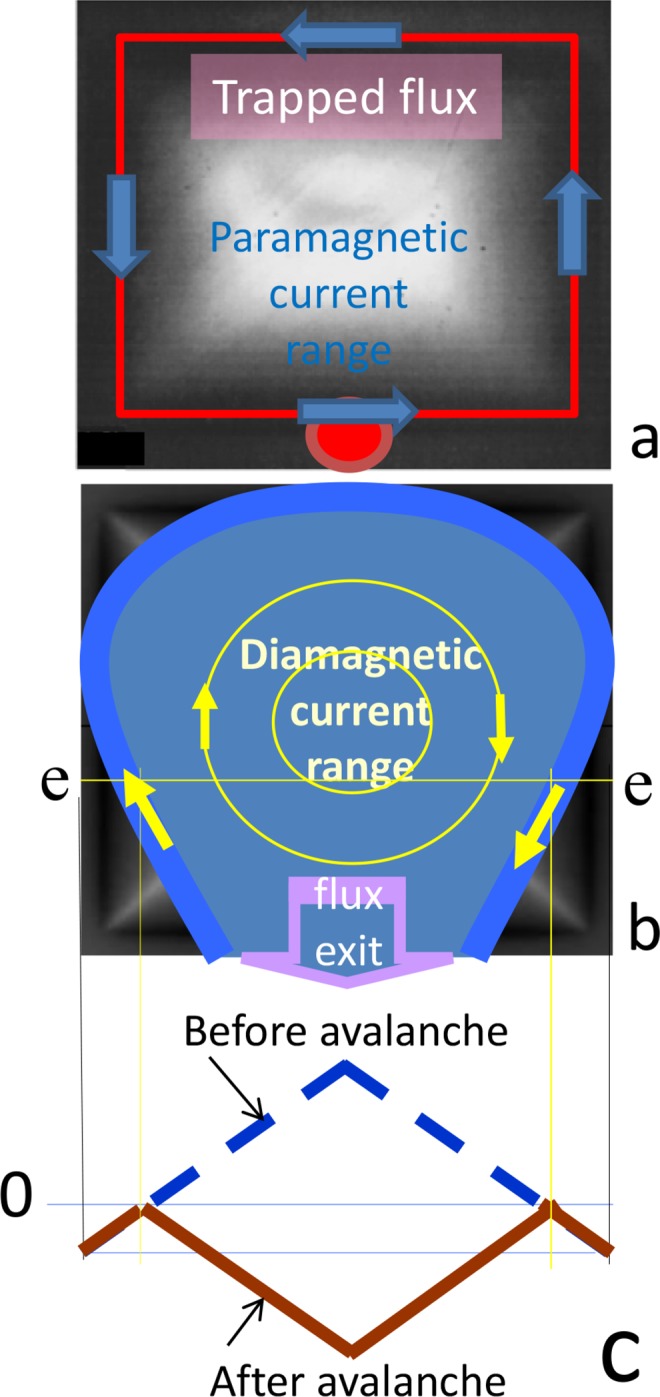
(**a**) Image of trapped flux distribution, i.e., visualization of “dome-like” structure of a YBa_2_Cu_3_O_7−*δ*_ square shaped film at 4.2 K^34^. (**b**) Drawing of the flux exit at avalanche. (**c**) Schematic view of induction profile evolution at avalanche in the Bean model, drawn along the e-e section of panel (b).

A description of a possible magnetic moment inversion of a superconductor in the case of an avalanche can be given as follows. The distribution of the trapped flux (“dome-like” structure) in a YBa_2_Cu_3_O_7−*δ*_ square-shaped film at 4.2 K is given in Fig. 3(a) (after^34^). The maximum induction values correspond to the brightest places. Absolutely black places correspond to zero normal component of induction *B*_n_. A similar picture of induction in the capture of magnetic flux is expected for the studied sample of the cubic form.

The “dome-like” structure of the induction is produced by the superconducting critical current *J*_c_, the direction of which is indicated by the arrows in Fig. 3(a). The magnetic moment of a superconducting sample fully penetrated by the magnetic flux is determined by integration of the critical current in the volume *V* of sample: $\overrightarrow{M}=\frac{1}{2}{\int }_{V}\,[\overrightarrow{r}\times {\overrightarrow{J}}_{c}]dV$.

To invert the magnetic moment, the change of critical current direction to the opposite one during flux avalanche is required, and the avalanche “crater”, formed by the flux exit, must occupy practically the entire area of the sample.

The construction of a scheme for inverting the magnetic moment in the case of an avalanche, presented on Fig. 3(b), was facilitated by the data of another researchers^27,28,33,35^, where the magneto-optical technique was used to study the transformation of the magnetic field induction pattern on the surface of a superconducting disk during the avalanche dynamics. Analysis of these data allowed us to draw the following conclusions: (a) the avalanche of the exit of trapped flux originates near the edge of the sample and its front propagates inward in the form of an arc, oriented by the convexity toward the center^27^; (b) the value of the critical current at the front of the avalanche, in dynamic conditions, is close to its quasi-static value, and the direction is opposite to that of current near sample edges, which ensures the capture of the magnetic flux^27,33^; (c) the avalanche front, encountering an obstacle, is deformed in accordance with its shape^35^.

Taking into account the aforementioned process, the transformation of the profile in the case of an avalanche can be represented schematically as shown in Fig. 3. Suppose that the avalanche of the flux exit, originated near the surface, for example at the point on the “dome-like” structure (Fig. 3(a), the point at the bottom of the picture), and its front reach almost the edges of the sample, as shown in Fig. 3(b). On the boundary of the avalanche, a critical state layer with a current of diamagnetic direction opposite to the current providing magnetic flux capture emerged^33^. This current realizes the inversion of the magnetic induction profile in the central part of the avalanche spot (creates a “crater”, Fig. 3(c)). Apparently, in our case the “crater” from the exiting flux acquired a shape close to a square one, similar to the geometry of the sample. In this case, the area over which the critical current flows, i.e. the current of the diamagnetic direction, is equal to the area of the superconductor with the trapped magnetic flux prior the avalanche. In this case, the inversion of the magnetic moment during the avalanche is almost complete.

Opposite direction of the captured moment in position 1 (as well as in position 4) of hysteresis loop (Fig. 2(c)) and of the external field should be noted. Such a state is energetically unfavorable, and the resulting interaction tends to unfold the moment of the superconductor in the direction of the field, promoting of the avalanche inversion.

We believe, a number of factors in our experiment might have contributed to the observation of the inversion of the superconductor’s critical state. The size of the avalanche spot depends on the stored energy of the superconductor just prior the emergence of instability of the critical state, the amount of heat dissipation to the environment^17^, dissipative losses during the avalanche flux dynamics, etc^14,36–38^. These factors were optimized due to the high critical parameters of the material at a low experimental temperature and quasi-adiabatic boundary conditions for the sample. An important role of the low pressure of helium gas in the surrounding medium, creating conditions similar to a vacuum sample bath, is noted.

The conditions of our experiment are to a certain extent exotic. In practice, the levitation processes are applied on spacecraft, where some of these exotic conditions of our experiment may be present. In addition, modern technologies make it possible to obtain a trapped field of 17 T at 29 K in the bulk superconductors^39,40^. In such materials, giant critical currents and thermomagnetic avalanches with moment inversion can be expected at higher temperatures than in our experiment. Instabilities can be stimulated by a random magnetic field pulse or, for example, by a push (shock) arising from the movement of one of the objects involved in levitation.

It should be added that magnetization inversion was registered locally (the local surface self-field) earlier in conventional NbTi superconductors and in textured YBa_2_Cu_3_O_7−*δ*_ by a Hall probe^30,41^, a Hall sensor array^31,32^, and a strain gage (magnetostriction)^42^, as a result of partial flux jumps, for the glued sensor located in the region of the avalanche spot. In our experimental conditions, an avalanche spot covers almost the entire sample. Therefore, here, the inversion of the magnetization at flux avalanches in the *integral* characteristics is discovered.

## Conclusions

The experiment on a bulk YBa_2_Cu_3_O_7−*δ*_ single crystal has revealed a new dynamical property of the critical state of type-II superconductors under a magnetic field, i.e., giant jump of magnetic moment, at external magnetic field close to zero, from one metastable state on the hysteresis loop to the state with opposite magnetic moment direction, as a result of thermomagnetic avalanches. Using the critical state concept, a model of the magnetic moment inversion was presented. Importantly, knowledge of conditions of the appearance of such a phenomenon is crucial for applications of bulk superconductors as “permanent” magnets, for example, in superconducting levitation devices, etc.
